# *CDH1* mutations in gastric cancer patients from northern Brazil identified by Next- Generation Sequencing (NGS)

**DOI:** 10.1590/1678-4685-GMB-2014-0342

**Published:** 2016-05-13

**Authors:** Antonette El-Husny, Milene Raiol-Moraes, Marcos Amador, André M. Ribeiro-dos-Santos, André Montagnini, Silvanira Barbosa, Artur Silva, Paulo Assumpção, Geraldo Ishak, Sidney Santos, Pablo Pinto, Aline Cruz, Ândrea Ribeiro-dos-Santos

**Affiliations:** 1 Laboratório de Genética Humana e Médica (LGHM), Instituto de Ciências Biológicas, Universidade Federal do Pará (UFPA), Belém, PA, Brazil; 2 Rede de Pesquisa em Genômica Populacional Humana, Universidade Federal do Pará (UFPA), Belém, PA, Brazil; 3 Instituto Sírio-Libanês de Ensino e Pesquisa, Hospital Sírio-Libanês, São Paulo, SP, Brazil; 4 Laboratório de Polimorfismo de DNA - Instituto de Ciências Biológicas, Universidade Federal do Pará (UFPA), Belém, PA, Brazil; 5 Núcleo de Pesquisas em Oncologia, Universidade Federal do Pará (UFPA), Belém, PA, Brazil; 6 Hospital Universitário João de Barros Barreto, Universidade Federal do Pará (UFPA), Belém, PA, Brazil

**Keywords:** CDH1, germline mutations, HDGC, Gastric Cancer, NGS

## Abstract

Gastric cancer is considered to be the fifth highest incident tumor worldwide and the third leading cause of cancer deaths. Developing regions report a higher number of sporadic cases, but there are only a few local studies related to hereditary cases of gastric cancer in Brazil to confirm this fact. *CDH1 g*ermline mutations have been described both in familial and sporadic cases, but there is only one recent molecular description of individuals from Brazil. In this study we performed Next Generation Sequencing (NGS) to assess *CDH1* germline mutations in individuals who match the clinical criteria for Hereditary Diffuse Gastric Cancer (HDGC), or who exhibit very early diagnosis of gastric cancer. Among five probands we detected *CDH1* germline mutations in two cases (40%). The mutation c.1023T > G was found in a HDGC family and the mutation c.1849G > A, which is nearly exclusive to African populations, was found in an early-onset case of gastric adenocarcinoma. The mutations described highlight the existence of gastric cancer cases caused by *CDH1* germline mutations in northern Brazil, although such information is frequently ignored due to the existence of a large number of environmental factors locally. Our report represent the first *CDH1* mutations in HDGC described from Brazil by an NGS platform.

## Introduction

Gastric cancer was considered to be the fifth highest incident tumor in both genders worldwide in 2012 and the third leading cause of cancer deaths (8.8% of the total). Although East Asia represents the region of greatest gastric cancer mortality, high rates are also observed in both genders in Central and Eastern Europe and in Central and South America (Ferlay *et al.*, 2010). In Brazil, it was estimated that gastric cancer was the fourth most frequent in men and the fifth most common in women in 2014. In the northern region, gastric cancer is the second most common in men and the third most common in women (INCA, 2014).

Corso *et al.* (2012) stated that developing regions of the world have a greater number of sporadic cases. Although only a few local studies have addressed this question, their results suggest that this statement is also applicable to Brazil. The gene related to the gastric cancer predisposition syndrome (Hereditary Diffuse Gastric Cancer - HDGC) is *CDH1* which encodes the E-cadherin protein, responsible for cell adhesion in non-neural epithelial cells, among other functions. Germline mutations of this gene as a cause of familial cases of diffuse gastric cancer were first described in the Maori tribe in New Zealand, in which the existence of a genetic syndrome predisposing to gastric cancer was first detected (Guilford *et al.*, 1998, 1999; Blair *et al.*, 2013).

To date, *CDH1* mutations have been described in various populations around the world. Noteworthy are the descriptions of mutations associated with carcinogenesis of different tumor types, such as breast cancer, prostate cancer, and gastric cancer (Richards *et al.*, 1999; Ikonen *et al.*, 2001; Masciari *et al.*, 2007; McVeigh *et al.*, 2014). Germline *CDH1* mutations have been described in approximately 30-40% of familial cases and in a smaller proportion of sporadic cases (Kaurah *et al.*, 2007; Garziera *et al.*, 2013;). Although several mutations have been detected in distinct families, no hotspot has been characterized. To date, there is only one molecular description of individuals from Brazil (Moreira-Nunes *et al.*, 2014).

Little has been discussed regarding the ancestral origin of pathogenic mutations described in cases of diffuse gastric cancer. However, a population approach is important when dealing with rare disorders like HDGC, because specific mutations observed in a population can guide the testing approach in other individuals of the same group.

Given the high incidence of gastric tumors in Brazil, specifically in the state of Pará, it is of great importance to focus the attention of clinicians and researchers on genetic factors potentially associated with gastric cancer in this population.

## Subjects and Methods

### Subjects

The study included six individuals of which only two were related (cases 1 and 3). Among the patients, four (cases 1, 3, 4 and 5) matched the clinical criteria for HDGC of the International Gastric Cancer Linkage Consortium (Fitzgerald *et al.*, 2010) and two (cases 2 and 6) had early onset (< 40 years) diffuse-type gastric cancer (Fitzgerald *et al.*, 2010; Kluijt *et al.*, 2012). Five patients (cases 1 to 5) were from northern Brazil and one individual was from southeastern Brazil (case 6).

This study was approved by the Research Ethics Committee of Universidade Federal do Pará - Hospital João de Barros Barreto (protocol number 359.927), obeying the principles of the Declaration of Helsinki and Nuremberg Code. All individuals signed an Informed Consent form.

### Genotyping analysis

DNA extraction was performed with the PureLink Genomics™ Mini Kit(Life Technologies, Foster City, CA, USA) according to the manufacturer's protocol. Amplification of the coding regions of the *CDH1* gene was performed by PCR, with a total of 20 amplicons per patient. The amplicons of the five index individuals (Table 1) were sequenced on an Ion Torrent PGM™ platform (Life Technologies).

**Table 1:** Polymerase chain reaction primers used for amplification of *CDH1* promoter region and its 16 exons, their product size and flanking regions size.

Region	Forward and reverse primers	Product size (bp)	Flanking regions size (bp)
Promoter	5’ GAGAACTCAGTAAAGGGGCTGA 3’	853	5’– 108
	5’ ACTAAGACCTGGGATCAGAAAGG 3’		–
Exon 1	5’ CCATCTCCAAAACGAACAAAC 3’	752	–
	5’ GAACTTTCTTGGAAGAAGGGAAG 3’		3’ – 113
Exon 2	5’ CTAGGTCTTGAGGGGGTGACT 3’	486	5’ – 236
	5’ GTAAATTCCAAGGGGTGTCGT 3’		3’ – 135
Exon 3	5’ GTAAATTCCAAGGGGTGTCGT 3’	421	5’ – 80
	5’ CAACCCCTACAACACAAAATCA 3’		3’ – 117
Exon 4	5’ TCAAACTGTACACTGCCCACA 3’	347	5’ – 117
	5’ ATCCCAACACTGGGTCTTTTC 3’		3’ – 86
Exon 5	5’ TCTGTTTCTCTGGGAGGGATT 3’	383	5’ – 111
	5’ TCAAGTTAAGCTCCTCATGTGTTC 3’		3’ – 106
Exon 6	5’ GTCACCCTCACTTGGTTCTTTC 3’	280	5’ – 22
	5’ CCGTAGGAAGGATCAGCTTTAGT 3’		3’ – 111
Exon 7	5’ TTCTTTCTCCCCTAGCACTTTG 3’	436	5’ – 169
	5’ ACAACTGGCCTAGCAGGATTT 3’		3’ – 91
Exon 8	5’ CTTGGTTGTGTCGATCTCTCTG 3’	194	5’ – 103
	5’ GACCTTTCTTTGGAAACCCTCT 3’		3’ – 40
Exon 9	5’ ATGATCGCTCAAATACACTCCA 3’	429	5’ – 148
	5’ CTGCCAAAGCGAATCTACTTCT 3’		3’ – 99
Exon 10	5’ CATTGAAAGTCATGGCAGAAAC 3’	420	5’ – 142
	5’ GCTGCAAGTCAGTTGAAAAATC 3’		3’ – 33
Exon 11	5’ GCTTAAGCCGTTTTCAGCTACA 3’	303	5’ – 70
	5’ AACTCTTCCCTCCAAAAGAAGG 3’		3’ – 87
Exon 12	5’ CTAGACTTGGTCTGGTGGAAGG 3’	430	5’ – 79
	5’ GGAAGCAAGTATCAATGGAAGG 3’		3’ – 126
Exon 13	5’ AAGCAGCTCTGCTCTCTTCACT 3’	470	5’ – 122
	5’ CTCTTTCCCACATCAGCTAACC 3’		3’ – 120
Exon 14	5’ TCTGTGATAGCTGCTGCTTCTG 3’	294	5’ – 75
	5’ AGCTGTTTCAAATGCCTACCTCT 3’		3’ – 88
Exon 15	5’ AAGGCATCATCCAACCATAATC 3’	311	5’ – 100
	5’ TTTTTGACACAACTCCTCCTGA 3’		3’ – 67
Exon 16.1	5’ AAGTCTGGGTGCATTGTCGTA 3’	690	5’ – 110
	5’ AGCTGACTTCTCCCCTTCTTTT 3’		–
Exon 16.2	5’ CAGCACCTTGCAGATTTTCTTA 3’	840	–
	5’ CTAGTCAAGATGTGGCCAGACA 3’		–
Exon 16.3	5’ CAGTTGCTTTGCCCAAGATAG 3’	817	–
	5’ TAGCTTGAACTGCCGAAAAATC 3’		–
Exon 16.4	5’ GGTAGTGAGGATCTTGATTTGGA 3’	398	–
	5’ CCTCTTTCTCCACGTTTTGACT 3’		3’ – 90

### Next Generation Sequencing (NGS)

Each of the 20 amplicons, including the promoter region of *CDH1* and its 16 exons (Table 1), were combined into a single equimolar pool with a total of 100 ng of DNA in a final volume of 35 μL.

The fragmentation of samples for 200 bp sequencing was performed with the ION shear plus reagent kit (Life Technologies) followed by purification with Agencourt™ AMPure Reagent XP™ (Agencourt Bioscience Corporation, Beverly, MA, USA). The connection of barcode adapters and repairs were made with the following kits: Ion Plus kit fragment library (Life Technologies) and Ion Xpress Barcode Adapters 1-16 kits (Life Technologies) in each one of the individual samples.

After purification, the samples passed through size selection in an e-Gel size selection 2% agarose gel, from which a band of 200 base pairs was retrieved. The library was then amplified, purified and assembled in the same pool concentration for emulsion PCR with the Ion PGM 200 template reagent kit (Life Technologies).

The sequencing reaction was performed with the ION sequencing reagent kit (Life Technologies). The resulting data was aligned to the reference genome hg19 (available at http://genome.ucsc.edu) and the mutations were identified using the GATK v.2.6. Toolkit. The variants were filtered by low quality calling (less than 50X depth and homo-polymer runs) and analyzed using the Integrative Genomics Viewer software (IGV v.2.3) (Broad Institute; https://www.broadinstitute.org/igv).

### Validation

**Figure 1:**
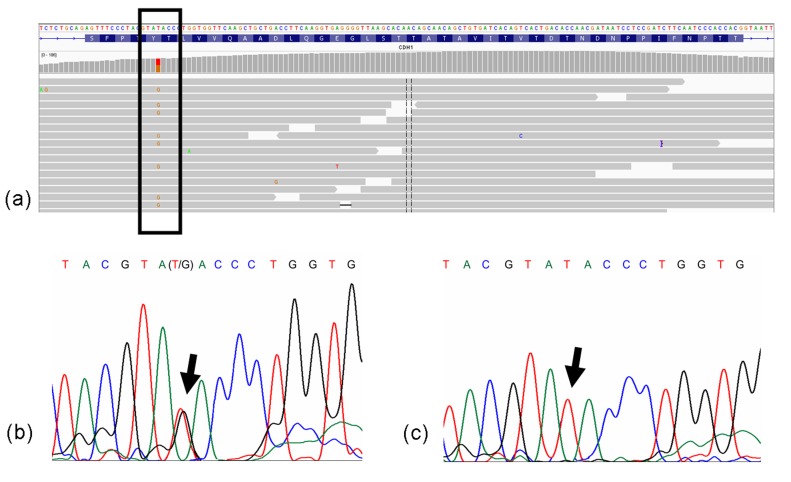
Molecular analysis presenting exon 8 *CDH1* mutation, c.1023T > G. (a) Integrative Genomics Viewer - IGV™ software result; (b) Sanger sequencing analysis of a patient; (c) Sanger sequencing analysis of a control case.

After identifying the familial mutation (index individuals), first-degree relatives were screened for the specific mutation (Figure 1) by Sanger sequencing. Although being a limited analyses for clinical purposes, every missense mutation detected had its potential pathogenicity tested using Polyphen-2 (Adzhubei *et al.,* 2010) and SIFT (Ng and Hennikoff, 2003) for predicting functional effects of human nsSNPs.

The main detected mutations were investigated by Sanger sequencing of 100 samples from the local population of Belém, PA, Brazil, for comparative purposes. Additionally, all populational data for mutations were checked using the 1,000 genomes project data (McVean *et al.,* 2012).

For each exon, the sequencing reaction was performed with 1 μL of purified PCR product of each exon, 0.5 μL of the reverse specific primer, 0.5 μL of Big Dye Terminator V3.1 Cycle Sequencing Kit (Life Technologies), and 3.0 μL of SaveMoney buffer to a final volume of 5 μL. The thermocycling reaction proceeded as follows: 95 °C for 2 min, followed by 35 cycles of 95 °C for 30 s, 60 °C for 20 s and 72 °C for 2 min.

After thermocycling, the product was prepared for sequencing in an ABI 3130 automatic sequencer (Life Technologies). The sequence information was interpreted by ABI Analysis Software™. The electropherograms were analyzed using the ChromasPro1.49 software and compared with the reference sequence obtained from GenBank (NM_004360.3).

### Analysis of genetic ancestry

All five index cases had their genetic ancestry tests performed using 48 INDEL informative markers of ancestry (American Indians, Europeans and Africans), following the methodology previously described by Santos *et al.* (2010). Three multiplex PCR reactions were performed, each with 16 markers, followed by electrophoresis on an ABI-PRISM 3130 sequencer and analysis using GeneMapper ID v. 3.2 (Life Technologies). The individual proportions of European, African and Amerindian genetic ancestry were estimated using the STRUCTURE software v.2.3.3, assuming three parental populations (Europeans, Africans and Amerindians) and running with a 200,000 burn-in period and 200,000 Markov Chain Monte Carlo repetitions after burning.

## Results

Results of the molecular analyses of the *CDH1* gene are summarized in Table 2. Among five probands, we detected *CDH1* germline mutations in two cases (40%). Case 1 exhibited a heterozygous *CDH1* exon 8 germline mutation c.1023T > G. This patient is member of a classic HDGC family. Case 3, who belongs to the same family, confirmed the presence of the c.1023T > G mutation.

**Table 2:** Germline mutations and polymorphisms detected in *CDH1* exons in hereditary diffuse gastric cancer in Brazil.

	Exon	*CDH1* mutation	Type of mutation	Prediction	Ancestry panel			Previous References	
					EUR	AME	AFR	Tumor type	Origin
Case 1*	8	c.1023T>G/p.Tyr341*	Nonsense	Pathogenic	0,517	0,334	0,149	Gastric cancer ^(McVean *et al*., 2012)^	Unavailable
	13	rs1801552	Silent	–				Populational studies ^(Adzhubei *et al*., 2010)^	Unavailable
		c.2076T>C/p.Ala692=							
Case 2	12	rs33935154	Missense	Benign**	0,520	0,167	0,313	Endometrial cancer ^(Risinger *et al*., 2010)^	Unavailable
				(SIFT score 0.19)				Sporadic diffuse gastric cancer ^(Guilford *et al*., 2010)^	African-American
				(PSIC score 0.04)				Early onset diffuse gastric cancer ^(Risinger *et al*., 1994)^	African-American
		c.1849G>A/p.Ala617Thr						Populational studies ^(Adzhubei *et al*., 2010^	Unavailable
	13	rs1801552	Silent	–					
		c.2076T>C/p.Ala692=							
Case 3*	8	c.1023T>G/p.Tyr341*	Nonsense	Pathogenic	0,391	0,385	0,224	Gastric cancer ^(McVean *et al*., 2012)^	Unavailable
	13	rs1801552	Silent	–				Populational studies ^(Adzhubei *et al*., 2010)^	Unavailable
		c.2076T>C/p.Ala692=							
Case 4	13	rs1801552	Silent	–	0,392	0,356	0,252	Populational studies ^(Adzhubei *et al*., 2010)^	Unavailable
		c.2076T>C/p.Ala692=							
Case 5	13	rs1801552	Silent	–	0,511	0,399	0,090	Populational studies ^(Adzhubei *et al*., 2010)^	Unavailable
		c.2076T>C/p.Ala692=							
	16	rs2229044	Silent	–				Populational studies ^(Adzhubei *et al*., 2010)^	
		c.2634C>T/p.Gly878=							
Case 6	13	rs1801552	Silent	–	0,656	0,186	0,158	Populational studies ^(Adzhubei *et al*., 2010)^	Unavailable
		c.2076T>C/p.Ala692=							

* Individuals from the same family

** Pathogenicity prediction evaluated by SIFT (sorting intolerant from tolerant) and Polyphen2 (PSIC) softwares.

**Figure 2:**
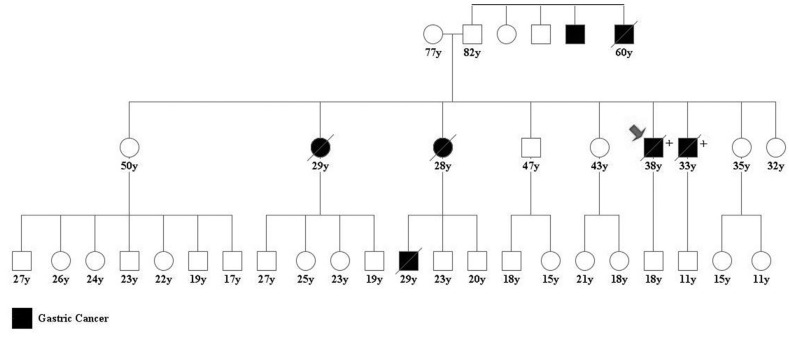
Pedigree of the *HDGC* Northern Brazilian family described in this paper, red arrow showing the index case (case 1); (+) represents individuals with molecular analysis showing the *CDH1* c.1023T > G mutation.

Figure 1a presents the molecular analysis of the *CDH1* germline mutation c.1023T > G by Integrative Genomics Viewer (IGV v.2.5) software, which was validated by Sanger sequencing (index case 1; Figure 1b). In a sample of 100 individuals from the local population of Belém, PA (Brazil) no instances of the mutation were found (Figure 1c). This mutation was identified as familial by analyzing first-degree relatives of cases 1 and 3 by Sanger sequencing. The family pedigree with 46 individuals is presented in Figure 2. Ten of the family members were tested for the c.1023T > G mutation and all exhibited the heterozygous mutation.

**Figure 3:**
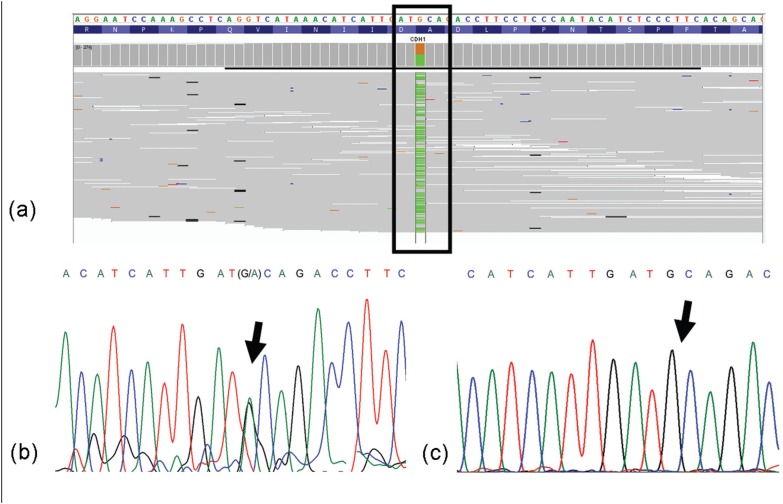
Molecular analysis presenting exon 12 *CDH1* mutation, c.1849T > G. (a) Integrative Genomics Viewer - IGV™ software result; (b) Sanger sequencing analysis of the patient; (c) Sanger sequencing analysis of a control case.

The molecular analysis of index case 2 revealed the *CDH1* germline mutation exon 12 c.1849G > A in heterozygosis (Table 2), which was confirmed by Sanger sequencing. This patient exhibited early-onset gastric adenocarcinoma (by 28 years of age) without any other similar case in the family. Figure 3a presents the molecular analysis for the *CDH1* germline mutation c.1849G > A by IGV v.2.3. Similar to index case 1, this mutation was not identified in the sample from local population (Figure 3c).

Previous population studies performed by the 1000 Genomes Project (McVean *et al.*, 2012) did not detected the c.1023T > G mutation, but described the c.1849A mutation as almost exclusively African, with an allele frequency of 0.045 and overall database frequency (MAF) of 0.01.

NGS alignment presented two INDEL variations with good quality (more than 50X depth) in heterozygous state among all individuals: c.1649delG and c.2218delC located in exon 11 and exon 14, respectively. Such kind of frame shift mutations would cause drastic damage for the final protein, and to us they seem to be incompatible with the biological and epidemiological background of gastric cancer. These variants probably represent sequencing errors of the platform chemistry, since they were found in highly repetitive regions. Thus, we choose to validate all our findings by Sanger sequencing.

No pathogenic *CDH1* germline mutation was confirmed in the index cases 4, 5 and 6, and only polymorphisms were observed (Tables 2 and 3).

**Table 3:** Known polymorphisms detected in *CDH1* introns and flanking regions.

CDH1 region	Cases					
	Case 1*	Case2	Case 3*	Case 4	Case 5	Case 6
5' flanking region						
rs7194355 (C > A)	*wt*	A/A	*wt*	C/A	C/A	*wt*
rs35582463 (C > T)	C/T	*wt*	C/T	*wt*	*wt*	*wt*
rs33945903 (C > T)	C/T	*wt*	C/T	*wt*	*wt*	*wt*
rs5030625 (GA > G)	G/G	G/G	G/G	G/G	G/G	GA/G
rs3395334 (C > A)	C/A	*wt*	C/A	*wt*	*wt*	*wt*
rs16260 (C > A)	*wt*	A/A	A/A	C/A	C/A	*wt*
Intron 1						
rs3743674 (C > T)	T/T	T/T	*	T/T	T/T	*wt*
rs147838237 (C > CGCCCCAGCCCCGT)	hoz	hoz	*	hoz	hoz	*wt*
rs286579983 (T > C)	T/C	T/C	*	T/C	T/C	*wt*
rs12928281 (C > T)	C/T	C/T	*	C/T	C/T	C/C
Intron 6						
rs8059669 (A > C)	*	*wt*	A/C	*wt*	*	A/C
Intron 7						
rs34374107 (T > C)	*wt*	*wt*	*wt*	*wt*	*wt*	*wt*
Intron 9						
rs35423758 (C > T)	*wt*	*wt*	*wt*	C/T	*wt*	*wt*
rs339509003 (G > C)	*wt*	*wt*	*wt*	*wt*	*wt*	*wt*
Intron 12						
rs2276330 (T > C)	T/C	*wt*	*wt*	*wt*	T/C	*wt*
3'UTR						
rs1801026 (C > T)	C/T	*wt*	*wt*	*wt*	C/T	*wt*
rs8049282 (C > T)	C/T	*wt*	C/T	C/T	*wt*	*wt*
rs33956791 (C > T)	*wt*	*wt*	*wt*	*wt*	*wt*	*wt*
rs9282653 (G > A)	*wt*	*wt*	*wt*	*wt*	*wt*	*wt*
rs13689 (T > C)	T/C	*wt*	*wt*	*wt*	*wt*	*wt*
3′ flanking region						
rs8045438 (A > G)	G/G	G/G	G/G	G/G	G/G	G/G
rs181705992 (T > A)	*wt*	*wt*	*wt*	*wt*	*wt*	*wt*
rs17690554 (C > G)	C/G	*wt*	*wt*	*wt*	*wt*	*wt*

*wt*, wildtype

* no call; *hoz*, mutant homozygous

When analyzing the genetic ancestry contribution, index case 1 exhibited a 52% European, 33% Amerindian and 15% African contribution. Index case 2 exhibited a 52% European, 17% Amerindian and 31% African contribution. These results are presented in Table 2.

Besides the exonic alterations, NGS was also able to detect intronic modifications. The ones already described as polymorphisms and registered in NCBI dbSNP are shown in Table 3.

## Discussion

The *CDH1* germline mutation of index case 1 (c.1023T > G) had been previously described in New Zealand patients, with only few clinical details available (Guilford *et al.*, 2010). It is responsible for the introduction of a premature stop codon at position 341 of the E-cadherin (p.Y341*) protein and is therefore pathogenic. This region encodes the second cadherin domain that is normally located in the extracellular portion of the protein and is essential for its juxtacellular adhesion function. Significant levels of mature protein cease to be translated in the presence of this mutation. Given the typical genetic admixture in Brazil, evidenced by studies of population ancestry (*e.g.* Santos *et al.*, 2010), it was not possible to associate ethnicity with the presence of the c.1023T > G mutation. However, the absence of this mutation in 100 individuals of the local population (Belém, PA, Brazil) and in the 1,000 genome project dataset (McVean *et al.*, 2012) puts in evidence that the mutation must be a rare mutation rather than a polymorphic variant.

Case 2 exhibited the c.1849G > A mutation previously detected by Risinger *et al.* (1994) in a tissue sample of endometrial cancer, and by Ascano *et al.* (2001) in patients with diffuse gastric cancer. Subsequently, Suriano *et al.* (2003) demonstrated the functional inactivation *in vitro* of c.1849G > A in cases of early onset gastric cancer like the present case. Supported by these studies, the pathogenicity of this mutation is described in the NCBI SNP database (http://www.ncbi.nlm.nih.gov/projects/SNP) under the code rs33935154.

However, analysis performed by Polyphen-2 (Adzhubei *et al.*, 2010) and SIFT (Ng and Hennikoff, 2003) software suggest low pathogenicity for this mutation (PSIC score 0.04; SIFT score 0.19), since different species present substitutions in the same protein position (p.617). Population studies performed in the 1,000 genomes project (McVean *et al.*, 2012) described the overall frequency (MAF) of allele c.1849A as 0.01. In the same database, the presence of this mutation is almost exclusive of African populations, with an allele frequency increasing to 0.045.

This divergent information can be explained under at least four hypotheses: (i) the mutation has incomplete penetrance; (ii) the mutation is not truly pathogenic, given the high frequency in African populations, where there is no significant increase in the HDGC case number or diffuse type gastric cancer at a young age; although scientific data on this subject are minimal; (iii) the mutation is pathogenic for other population groups but not among Africans, given its local frequency of 4.5%; (iv) the mutation is only pathogenic in the presence of other genetic and/or epigenetic factors not yet studied. As there is no study testing this mutation in African patients with diffuse gastric cancer, all hypotheses above must be considered.

In case 2, it is likely that the mutation occurs as an effect of the patient's African ancestry contribution of 31%. Interestingly, Suriano *et al.* (2003) described the c.1849A mutation in two African-American-unrelated cases. The geographical origin of these two cases was not available.

Regarding the polymorphisms described in this paper, we highlight the exonic rs1801552 and the intronic: rs13689, rs16260 and rs17690554, already analyzed by Zhan *et al.* (2012) in a case-control study without any difference detected between genotypes in gastric cancer patients and control group.

Previous studies on rs16260 and also on rs1801026 have called attention to the possibility of being markers for genetic susceptibility to cancer (Wang *et al.*, 2007; Li *et al.*, 2011). But conflicting results were shown when comparing different populations (Li *et al.*, 2012). Additional population studies of Brazilian subjects from different geographic regions of the country should be performed to find out whether these polymorphisms can provide useful susceptibility information in our country.

Recent studies of *CDH1* with NGS focused mostly on hereditary breast cancer (Castera *et al.*, 2014; Yang *et al.*, 2015). Dang *et al.* (2014) studied *CDH1* mutations in gastric cancer tissues with NGS, aiming to clarify their particular pathogenesis. All of these studies were performed on different sequencing platforms than the one used here, and concluded that NGS technology was an excellent method for their investigations.

Regarding the mutations c.1649delG and c.2218delC detected by NGS in all cases, it is unlikely that they represent low mosaicism with clinical significance, as they presented large read depth and calling quality in every case analyzed. Rather, they seem to be due to a sequencing error of a highly repetitive region of the genome. Although NGS techniques generate highly reliable data, they still can produce several miss calling due to specifics of their chemistry and software limitations. Thus, a critical analysis is necessary when interpreting variant reports, and suspicious results must be double checked by techniques such as Sanger sequencing or others.

Furthermore, although three of the six index cases presented no pathogenic mutation, the proportion of diffuse gastric cancer cases with mutations detected is similar to previously reports in the literature (40%; Kaurah *et al.*, 2007; Garziera *et al.*, 2013). Quite possibly, other genes or epigenetics factors may be the cause behind these undefined cases.

## Conclusion

The mutations described in this paper demonstrate the existence of gastric cancer cases caused by *CDH1* germline mutations in an endemic region of gastric cancer (northern Brazil), and such information is frequently ignored due to the significant number of environmental factors present.

The presence of the c.1849G > A mutation, a mutation almost African-exclusive, demonstrates the importance of considering ancestry and ethnicity when studying genetic disorders.

These *CDH1* germline mutations (c.1849G > A; c.1023T > G) are the first described in association with HDGC and early onset gastric cancer from Brazil revealed by a Next-Generation Sequencing platform. Larger studies that examine the frequency of gastric cancer cases associated with an abnormal *E-cadherin* gene will be of great value to determine the true importance of this genetic factor for gastric cancer in this area.
